# Efficacy of a Herbal Toothpaste During Active Periodontal Treatment: A Clinical Study

**DOI:** 10.3390/dj12120378

**Published:** 2024-11-22

**Authors:** La-ongthong Vajrabhaya, Supranee Benjasupattananan, Kraisorn Sappayatosok, Vittawin Dechosilpa, Suwanna Korsuwannawong, Papatpong Sirikururat

**Affiliations:** 1 College of Dental Medicine, Rangsit University, Pathumthani 12000, Thailand; la-ongthong.v@rsu.ac.th (L.-o.V.); supranee.b@rsu.ac.th (S.B.); kraisorn.s@rsu.ac.th (K.S.); vittawin@rsu.ac.th (V.D.); 2 Research Office, Faculty of Dentistry, Mahidol University, Bangkok 10400, Thailand; suwanna.aut@mahidol.ac.th

**Keywords:** *Aloe vera*, herbal product, toothpaste, periodontitis, non-surgical periodontal treatment

## Abstract

**Backgound/Objectives:** This study investigated the efficacy of a herbal toothpaste containing *Aloe vera* (test group) compared with a sodium bicarbonate toothpaste (active control group) and a standard toothpaste (benchmark group) on periodontitis treatment outcomes. **Methods:** Fifty-four periodontitis patients were randomly allocated into three groups. The patients received mechanical instrumentation and instruction on oral hygiene using a toothbrush with the toothpastes and dental floss. The patients were evaluated at baseline (T0), week 4 (T1), and week 12 (T2) after complete scaling and root planing. During the visits, the plaque score (PS), bleeding on probing (BOP), probing depth (PD) and clinical attachment level (CAL) were assessed and analyzed. **Results:** The comparison groups had similar PS and BOP means at baseline. At T1 and T2, both scores were reduced; however, there was no significant difference in PS among the three groups. A significant reduction in BOP among the groups was observed (*p* < 0.01) at T1. The PDs in all groups were significantly reduced after treatment. The CAL reduction was greater in the test group compared with the benchmark and the active control group. Furthermore, there was no significant difference in the mean CAL among time points in the benchmark and the active control groups. **Conclusions:** The herbal toothpaste containing *Aloe vera* significantly decreased gingival inflammation, PD, and CAL over the standard and active control toothpaste in periodontitis patients during active periodontal treatment.

## 1. Introduction

Periodontitis is a multi-factorial disease that affects the majority of the world’s population. Key periodontal pathogens in the supra- and subgingival dental biofilm and periodontal tissues interact with the host immune response via innate and adaptive immunity. This results in connective tissue and alveolar bone destruction and can lead to tooth loss [1,2]. To arrest progressive attachment loss, the systemic and local risk factors associated with periodontitis must be controlled. Removing the dental plaque and deposits by scaling and root planing (SRP) during the active periodontal treatment is imperative [3]. Moreover, oral hygiene care in the strict supportive periodontal treatment demonstrated long-term stable periodontal status [4,5]. However, the long-term success of periodontal treatment is dependent upon the efficacy of periodontal instrumentation and patient’s cooperation to control the oral hygiene care.

Routine mechanical tooth cleaning by brushing with toothpaste is an effective method to reduce the pathogenic microbial load. To achieve this goal, toothpastes containing antibacterial and chemical agents, i.e., chlorhexidine, triclosan, and metal salts, have been evaluated [6]. Presently, the awareness of the minimal toxicity and less harmful effects of herbal toothpastes has increased. *Aloe vera* (*A. vera*) is a medicinal plant that is commonly used to treat acute or chronic wounds. It is a non-toxic substance and significantly increases fibroblast cell migration [7]. Additionally, the polysaccharides in *A. vera* gel reduced the bacterial load by promoting phagocytosis to eradicate the microbes [8]. A toothpaste containing *A. vera* demonstrated an antimicrobial effect on oral microorganisms, such as *Streptococcus mutans* and *Candida albicans* [9,10]. Moreover, a significant reduction in plaque accumulation from a mouth rinse containing *A. vera* was also observed [11]. The *A. vera* extract treatment also resulted in a significant reduction in glutathione, superoxide dismutase, catalase, glutathione peroxidase, and glutathione S-transferase in the liver and kidney of diabetic rats, demonstrating the antioxidant effect of *A. vera* gel extract [12]. Recently, a herbal toothpaste containing *A. vera* and other herbal products was analyzed in an in vitro study. It significantly increased gingival fibroblast cell migration and showed greater *Porphyromonas gingivalis* biofilm inhibition [13]. A toothpaste with antibacterial and enhanced healing effects could be an alternative option for oral care that might provide oral health benefits during periodontal disease treatment. However, the clinical efficacy of the herbal toothpaste containing *A.vera* in periodontitis treatment has not been determined.

The objective of the study was to evaluate the effect of a herbal toothpaste on PS, BOP, PD, and CAL during active periodontal treatment in non-surgical periodontal treatment (NSPT) patients. The null hypothesis was that the herbal toothpaste containing *A. vera* did not significantly decrease BOP, PD, and CAL in periodontitis patients during SRP compared to the control and the benchmark toothpaste.

## 2. Materials and Methods

### 2.1. Study Design and Population

**Figure 1:**
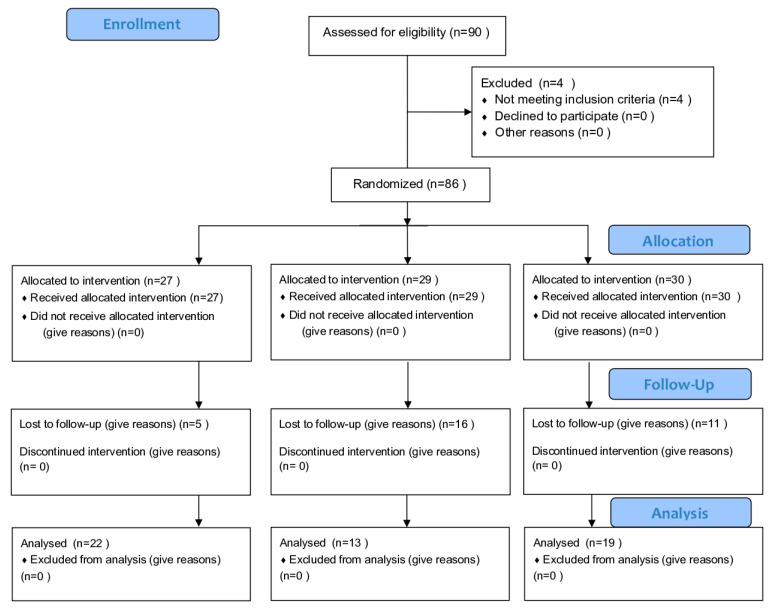
RCT flow diagram.

The protocol for the human experiment was approved by the Ethical Committee of the Research Institute of Rangsit University (Project number RSUERB2020-011). The study protocol was approved by the Thai Clinical Trials Registry on 16 May 2024 https://www.thaiclinicaltrials.org/show/TCTR20240516001 (accessed on 14 May 2024) (clinical trial registration number TCTR20240516001) (Figure 1).

The study was performed at the College of Dental Medicine, Rangsit University, Pathumthani, Thailand. The patients were required to have at least 4 permanent teeth with a periodontal pocket depth greater than 4 mm and radiographic alveolar bone destruction, diagnosed as periodontitis according to AAP/EFP2018. Furthermore, the patients had to (1) have a full-mouth plaque score > 40%, (2) exhibit > 30% bleeding on probing, (3) be between 20 and 70 years old, and (4) provide informed consent. The exclusion criteria comprised (1) having been diagnosed with dental-plaque-induced gingivitis or non-plaque-induced gingival lesions, (2) NSPT in the preceding 12 months, (3) orthodontic treatment within 12 weeks, (4) periodontal surgery in the preceding 12 weeks, (5) ongoing treatment with antimicrobials and/or anti-inflammatory medication, (6) pregnant or lactating, (7) smokers or excessive drinkers, and (8) a history of an allergic reaction to the toothpastes. Informed consent was obtained from all patients and/or their legal guardian(s). The gingival index was determined as the primary outcome of the study to calculate the sample size [10]. The group sample sizes were determined by G*power analysis version 3.1 with 90% test power and a significance level of 0.05. The total calculated sample size for all experimental groups was 42. The patients were arbitrarily allocated into one of three groups via simple randomization using a random number generator.

### 2.2. Intervention

The study involved a double-blinded randomized parallel-group comparison between the following three toothpastes: (1) herbal toothpaste containing the following active ingredients: *A. vera*, sodium chloride, mangosteen peel, whole *Hydrocotyle* plant, *Clinacanthus nutans*, orange jessamine leaf extract and toothbrush tree, as displayed in Table 1 (Twin Lotus Co., Ltd., Bangkok, Thailand) (test group); (2) sodium bicarbonate toothpaste (Parodontax^®^, Glaxo-SmithKline (Thailand), Ltd., Bangkok, Thailand) (active control group); and (3) standard toothpaste containing sorbitol, glycerin, calcium carbonate, and sodium lauryl sulfide (benchmark group).

All patients received an NSPT and oral hygiene instruction using a soft-bristle toothbrush (Colgate^®^, Colgate-Palmolive (Thailand), Ltd., Bangkok, Thailand) with one of the aforementioned toothpastes and dental floss (Colgate^®^, Colgate-Palmolive (Thailand), Ltd., Bangkok, Thailand) twice daily during the study. All patients were evaluated at baseline (before the treatment), T0; 4 weeks after complete full-mouth SRP, T1; and 12 weeks after SRP (end of the follow-up), T2. At the baseline and follow-up examinations, the following parameters were evaluated:

#### 2.2.1. Plaque Score (PS)

The teeth were stained with an erythrosine dye disclosing agent. The presence or absence of a continuous biofilm at the cervical third of the facial, lingual, and proximal surfaces of each tooth was determined. The percentage of tooth surfaces with dental biofilm was calculated for each patient [4].

#### 2.2.2. Bleeding on Probing (BOP)

The presence or absence of bleeding in six gingival areas around each tooth (mesio-facial, midfacial, disto-facial, disto-lingual, midlingual, and mesio-lingual) was assessed after probing. The percentage of gingival bleeding areas relative to the total number of gingival areas present was determined [4].

#### 2.2.3. Probing Depth (PD) and Clinical Attachment Level (CAL)

The Williams probe was used to measure PD (the distance from the gingival margin to the apical portion of the gingival sulcus) (mm) and CAL (the distance from the cementoenamel junction to the apical portion of the gingival sulcus) (mm) in six units around each tooth. The means of full-mouth PD and CAL were calculated.

### 2.3. Statistical Analysis

Because the data in each group were not normally distributed, the Kruskal–Wallis test was used to compare the evaluated parameters’ means after using the different toothpastes among the three comparison groups. The Bonferroni correction for multiple tests was utilized to determine the differences between the means within each group at different visits for PS and BOP. The Friedman test was performed to determine the differences between the means within each group at different visits for PD and CAL. The significance level was set at *p* < 0.05. Data analysis was performed using the Statistical Package for the Social Sciences version 18.0 for Windows (SPSS, Inc., Chicago, IL, USA).

## 3. Results

A total of 54 qualified patients agreed to join the study and were randomized into 3 groups (22 in the test group; 13 in the active control group; 19 in the benchmark group). The number of males and females in each group and mean age in each group are shown in Table 2. The age range of the patients was 34–70 years, and most were male (63%). There was no report of adverse side effects or allergic reactions from using any of the toothpastes. The full-mouth PS, BOP, PD, and CAL means at different time points were not significantly different among the groups at baseline (Table 3 and Table 4).

The comparison groups had similar mean PS and BOP at baseline (*p* > 0.05). At weeks 4 and 12, both PS and BOP were reduced. There was no significant difference among the three groups for PS. However, there was a significant reduction in BOP score in all groups (*p* < 0.01) at week 4. The active control group showed the lowest mean full-mouth BOP (13.83%) compared with the test and benchmark group: 17.52% and 21.03%, respectively. Moreover, there was no significant difference in BOP among the groups at 12 weeks (*p* > 0.05) (Table 3).

The PD and CAL means among the three comparison groups are shown in Table 4. There was no overall difference in PD and CAL among the groups at the beginning of the study. The PDs in all groups were significantly reduced in the same pattern after NSPT. In the test group, the PD decreased from 3.10 mm at baseline to 2.67 mm in week 4 and then to 2.63 mm in week 12. The reduction pattern was similar to that of the active control group, in which the PD decreased from 3.02 mm at baseline to 2.44 mm in week 4 and 2.52 mm at week 12. At week 4 and week 12, there also was a significant PD reduction in the benchmark group (*p* ≤ 0.001).

The changes in CAL in all groups at different time points are shown in Table 4. The CAL was similar among the groups at baseline. However, in the test group, the mean CAL significantly diminished from 2.71 mm at baseline to 2.30 mm at week 4 and 2.43 mm at week 12 (*p* < 0.05). A reduction was also observed in the benchmark and active control group from 2.63 and 2.71 mm to 2.34 and 2.62 mm, respectively; however, this change was not significant. The Kruskal–Wallis test revealed that the CAL reduction at the end of the study was greatest in the test group compared with the benchmark and active control group. In addition, there was no difference in the mean CAL between time points in the benchmark and active control groups.

## 4. Discussion

Periodontitis is a highly prevalent disease, and preventing its occurrence or recurrence is dependent on a patient’s plaque control. The goal of periodontitis treatment is to arrest progressive attachment loss, reduce pocket depths and control the systemic and local risk factors associated with periodontal diseases [3]. Mechanical tooth cleaning is mainly influenced by the willingness and dexterity of the individuals and by the design features of oral hygiene aids. Maintenance of an effective level of plaque control is difficult using conventional mechanical procedures and toothpastes but is required from a therapeutic point of view [4,5]. The latest meta-analysis showed the benefits of applying the topical agents, e.g., sulfonated phenolics gel, during NSPT between baseline and 3–6-month follow-up. The use of disinfectants and other products as adjuncts to NSPT was shown to improve the clinical and microbiological parameters compared to the conventional treatment [14]. The present study assessed whether the herbal toothpaste improved clinical gingival inflammation and periodontal parameters. The present study focused on the effect of the herbal toothpaste during NSPT. We found that the herbal toothpaste containing *A. vera* reduced BOP, PD, and CAL in untreated periodontitis patients.

A recent systematic review reported that herbal toothpaste had a superior effect compared with non-herbal toothpaste in short-term dental biofilm reduction [15]. In contrast, our study did not find a difference in plaque reduction among the three groups after short- and long-term observations. The significant plaque reduction among groups may be strongly influenced by the individualized oral hygiene instruction that was demonstrated chairside by the operator. Interestingly, there was an overall substantial significant difference in BOP reduction at week 4 among the study groups. At the end of follow-up, although there was no significant difference in BOP among the groups, the test group had the lowest mean full-mouth BOP (17.85%) compared with the active control and the benchmark groups. A longer follow-up time may be required to evaluate the long-term outcomes. This is consistent with the preexisting literature that found that the herbal toothpaste did not reduce BOP over the short term (4-week follow-up) and long term (12-week follow-up) compared with a non-herbal toothpaste [14]. Additionally, several studies found that the *A. vera* toothpaste reduced the gingival index in the treatment of gingivitis. No side effects were observed due to the use of this herbal toothpaste [10,16]. In the test group, the PD decreased from 3.10 mm at baseline to 2.63 mm at week 12. This reduction pattern was similar to that of the active control group and the benchmark group. However, in the test group, the mean CAL decreased from 2.71 mm at baseline to 2.43 mm at week 12, showing 0.28 mm in clinical attachment gain. A reduction was also observed in the benchmark and active control group; however, this change was not significant. The majority of the periodontitis cases in this study were defined as mild-to-moderate periodontitis, which had a mean full-mouth initial PD of 2.95–3.10 mm. The PD reduction and CAL gain were consistent with the literature that found that the two-year result of conventional periodontal therapy by scaling and root planing could achieve PD reduction and CAL gain of 0.40 and 0.29 mm, respectively [17].

For the past two decades, herbal-related research has remarkably affected oral health care, especially for oral lesions and periodontal and peri-implant disease treatment. Locally delivered applications predominate in dentistry, with toothpastes, mouth rinses, varnishes, and gels in numerous pharmaceutical forms being the most examined applications [18,19]. Most of the herbal toothpastes have antiplaque and anti-inflammatory effects [20,21,22]. However, there are a limited number of studies regarding the efficacy of herbal toothpaste as an adjunct to periodontal treatment. To the best of our knowledge, few studies have assessed the efficacy of *A. vera* as an adjunctive method in periodontitis treatment. The herbal toothpaste containing *A. vera* that was used in the present study has been studied in vitro. This formulation significantly increased human gingival fibroblast cell migration, which could have a positive effect on periodontal wound healing and regeneration. Furthermore, it demonstrated greater *Porphyromonas gingivalis* biofilm inhibition compared with the 0.12% chlorhexidine digluconate solution group [13]. The desensitizing efficacy of the herbal toothpaste was also determined. A study showed that after four weeks of use, this herbal toothpaste significantly reduced dentine hypersensitivity to the same extent as a 5% potassium nitrate toothpaste [23].

The results in this study indicate that the use of the herbal toothpaste had superior efficiency compared with regular toothpastes in treating periodontitis. A toothpaste that enhances wound healing is an alternative option for oral care that would provide oral health benefits for NSPT. However, the long-term effect of the herbal toothpaste containing *A. vera* should be investigated. Another study limitation is the sample size. Since the data in each group were not normally distributed, further research should employ both a greater sample size and a lengthier follow-up period.

## 5. Conclusions

In this study, it was documented that the herbal toothpaste containing *A. vera* significantly decreased gingival inflammation, PD, and CAL over the standard and active control toothpaste in periodontitis patients during NSPT.

## Figures and Tables

**Table 1:** List of the active ingredients in the study toothpastes.

Toothpaste	Active Ingredient	Full Scientific Species
Test group	*Aloe vera*	*Aloe vera* (L.) Burm. f.
	*Clinacanthus nutans*	*Clinacanthus nutans* (Burm.f.) Lindau.
	Orange Jessamine leaf	*Murraya Exotica* L.
	*Hydrocotyle*	*Centella asiatica* (L.) Urb.
	Toothbrush tree	*Streblus asper* Lour.
	Mangosteen peel	*Garcinia mangostana* Linn.
Active control group	Sodium bicarbonate	-
	Sodium fluoride	-
	Corn mint oil	*Mentha Arvensis*
	Purple coneflower	*Echinacea Purpurea*
	Krameria root extract	*Krameria Triandra*
	Chamomile extract	*Chamomilla Recutita*
	Sage oil	*Salvia officinalis*
Benchmark group	-	-

**Table 2:** The patients’ demographic data.

Number of Samples	Mean Age (years) ± SD
Test group (*n* = 22)	
15 Males	50.26 ± 7.24
7 Females	
Active control group (*n* = 13)	
9 Males	53.70 ± 8.98
4 Females	
Benchmark group (*n* = 19)	
10 Males	45.98 ± 6.67
9 Females	
Total *n* = 54	49.98 ± 7.63

SD: standard deviation.

**Table 3:** PS and BOP means among the three groups at different time points.

Percentage	Time	Test	Benchmark	Active Control	*p*-Value
PS	T0	44.66 ± 19.84	59.18 ± 22.93	55.99 ± 25.74	0.054
	T1	21.65 ± 11.27	21.55 ± 9.77	19.86 ± 7.48	0.118
	T2	26.20 ± 11.60	25.95 ± 14.20	35.62 ± 18.03	0.169
BOP	T0	47.87 ± 25.40	45.81 ± 24.21	42.18 ± 25.69	0.726
	T1	17.52 ± 14.66 ^a,b^	21.03 ± 19.97 ^a^	13.83 ± 10.61 ^b^	0.000
	T2	17.85 ± 13.47	29.13 ± 20.61	23.04 ± 18.93	0.223

PS: plaque score, BOP: bleeding on probing, ^a,b^ Indicates a significant difference between groups (*p* < 0.05).

**Table 4:** PD and CAL means among the three groups at different time points.

Mean (mm)	Time	Test Mean ± SD (mm)	Benchmark Mean ± SD (mm)	Active Control Mean ± SD (mm)
PD	T0	3.10 ± 0.44 *^,^**	2.95 ± 0.42 ^#,##^	3.02 ± 0.54^p,pp^
	T1	2.67 ± 0.48 *	2.40 ± 0.40 ^#^	2.44 ± 0.40^p^
	T2	2.63 ± 0.41 **	2.41 ± 0.36 ^##^	2.52 ± 0.31^pp^
	*p*-value	<0.001	<0.001	<0.002
CAL	T0	2.71 ± 1.08 ***	2.63 ± 0.90	2.71 ± 0.70
	T1	2.30 ± 0.10	2.33 ± 0.96	2.55 ± 0.70
	T2	2.43 ± 0.90 ***	2.34 ± 0.88	2.62 ± 0.72
	*p*-value	<0.050	0.211	0.758

PD: probing depth, CAL: clinical attachment level, *^,^**^,^*** Indicates a significant difference within the test group (*p* < 0.05). ^#,##^ Indicates a significant difference within the negative group (*p* < 0.05). ^p,pp^ Indicates a significant difference within the positive control group (*p* < 0.05).

## Data Availability

Data supporting the reported results can be obtained, on request, by writing to the corresponding author.
